# Deciphering the Anti-Tumor Mechanisms of Metformin Through Reprogramming of the Tumor Microenvironment

**DOI:** 10.3390/cells15131183

**Published:** 2026-06-29

**Authors:** Ting Zeng, Lemei Zheng, Jianxia Wei, Changning Xue, Qingqing Wei, Huizhen Xin, Zubing Wu, Ming Zhou, Mengna Li

**Affiliations:** 1 NHC Key Laboratory of Carcinogenesis, Hunan Cancer Hospital and the Affiliated Cancer Hospital of Xiangya School of Medicine, Central South University, Changsha 410078, China; 2 Cancer Research Institute, Xiangya School of Basic Medical Sciences, Central South University, Changsha 410078, China; 3 The Key Laboratory of Carcinogenesis and Cancer Invasion of the Chinese Ministry of Education, Central South University, Changsha 410078, China; 4 Hunan Key Laboratory of Oncotarget Gene, Hunan Cancer Hospital and the Affiliated Cancer Hospital of Xiangya School of Medicine, Central South University, Changsha 410013, China

**Keywords:** metformin, tumor microenvironment, anti-tumor effect, tumor progression, immunomodulation

## Abstract

**Highlights:**

**What are the main findings?**

- Metformin reprograms the tumor microenvironment (TME) by modulating immune, inflammation, vessel, matrix, metabolism and their interactions.
- Metformin exerts anti-tumor effects through reprogramming of the TME, thus regulating various pathways in tumor cells.

**What are the implications of the main findings?**

- Metformin-mediated TME reprogramming provides novel perspectives for understanding its mechanisms of anti-tumor effects.
- The combination of metformin and TME-targeted inhibitors may be a potential strategy in cancer therapy.

**Abstract:**

Metformin is a cornerstone medication for type 2 diabetes and exhibits anti-tumor activities. Previous studies have demonstrated that metformin suppresses tumor progression by regulating multiple signaling pathways, including the AMPK, PI3K/AKT/mTOR, and JNK pathways. However, most previous studies have focused on its direct effects on tumor cells, with limited attention to its effects in the TME. The TME constitutes a multifaceted ecosystem that drives tumor development and therapeutic resistance via physical barrier formation, immune evasion, and abnormal angiogenesis. In this review, we systematically summarize the impact and underlying regulatory mechanisms of metformin on distinct components of the TME. In addition, we discuss the individual and combined roles of metformin in immunity and inflammation, as well as vascular, matrix, and metabolic regulation. By elucidating the mechanisms of metformin-mediated TME reprogramming, we aim to provide new perspectives for understanding its anti-tumor effects and facilitating its clinical translation in cancer therapy.

## 1. Introduction

Metformin is a first-line therapeutic drug for type 2 diabetes mellitus [1]. Beyond its glucose-lowering properties, accumulating evidence has validated its anti-tumor activity, which effectively suppresses tumor cell proliferation, metastasis, stemness and chemoresistance in multiple malignancies [2,3,4]. Mechanistically, metformin activates canonical pathways, AMP-activated protein kinase (AMPK) and non-canonical pathways, including the HIF-1α [5], PI3K/AKT/mTOR [6] and JNK [3] signaling pathways. Despite some recent evidence, its regulatory roles in the tumor microenvironment (TME) remain poorly understood. The TME is a complex multicellular ecosystem comprising the extracellular matrix, cancer-associated fibroblasts, tumor vasculature, immune infiltrates and soluble mediators, components that critically modulate tumor progression, metastasis, immune evasion and metabolic reprogramming [7]. Intercellular crosstalk within the TME, such as immunosuppressive cytokine secretion and exosome-mediated signaling, further drives malignant development [8]. Notably, TME remodeling has emerged as a key mechanism underlying metformin’s anti-tumor efficacy: metformin inhibits glycolysis and lactate production to reverse TME acidification [9,10], and suppresses pathological angiogenesis to improve chemotherapeutic drug delivery [11]. Thus, clarifying metformin’s regulatory role in the TME is pivotal for deciphering its full anti-tumor mechanisms and promoting its clinical translation.

Herein, we systematically review the effects and molecular mechanisms of metformin on diverse TME components. This review aims to provide novel insights to expand its clinical applications in cancer therapy by dissecting its TME-mediated anti-tumor actions across cancers.

## 2. Metformin Mediates Precise Regulation in TME

### 2.1. Immune Cell Modulation by Metformin

Various recent pieces of evidence indicate that metformin plays a vital role in anti-tumor immunoregulation, making it a promising therapeutic agent for overcoming tumor-induced immunosuppression. Recent advancements utilizing single-cell RNA sequencing (scRNA-seq) and spatial transcriptomic analysis have revealed that metformin profoundly reshapes the immune landscape within the TME. Specifically, metformin treatment expands the proportion of CD8+ T cells and enhances their effector functions in colorectal cancer. Furthermore, it has been shown to increase the populations of T and B cells and promote the establishment of a distinct macrophage subpopulation in older people’s ovaries. The immunomodulatory mechanisms of metformin are detailed below.

**Figure 1:**
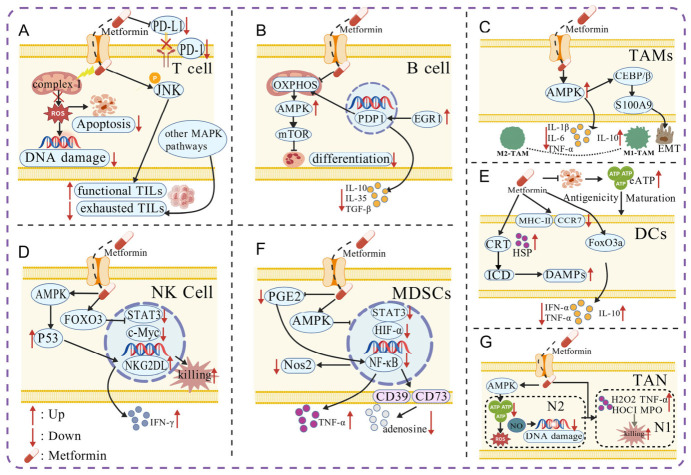
Metformin-mediated reprogramming of tumor immune microenvironment. (**A**). Metformin mediates the infiltration of functional T cells and protects them from apoptosis. (**B**) Metformin inhibits the differentiation of Bregs, thereby relieving their immunosuppressive effect. (**C**) The polarization of macrophages toward the M1 phenotype, which plays an anti-tumor role, is regulated by metformin. (**D**) Metformin enhances the ability of NK cells to recognize and kill tumors. (**E**) Metformin promotes dendritic cell maturation and antigen presentation. (**F**) The immunosuppressive function of MDSCs is weakened by metformin, which reduces tumor immune escape. (**G**) Metformin promotes neutrophils to the N1 phenotype, which plays an anti-tumor role.

T cells play a key role in anti-tumor immunity, and they directly dictate tumor progression. Thus, enhancing T cell function remains a critical strategy in tumor immunotherapy. Metformin has been demonstrated to bolster T cell function through multiple mechanisms to exert anti-tumor effects. It remodels the immune landscape of colorectal cancer with scRNA-seq analysis. Specifically, metformin treatment expanded the proportion of CD8+ T cells and potentiated their function [12]. First, metformin directly acts on mitochondrial complex I in CD8+ T cells to inhibit its activity, and this suppression reduces reactive oxygen species (ROS) production. It also protects CD8+ T cells from ROS-induced apoptosis and oxidative DNA damage, thereby enhancing their adaptability in the hypoxic microenvironment both in vitro and in vivo [13]. This mechanism is distinct from findings in C57/BL6 B16-F10 melanoma and MC38 colon adenocarcinoma models, where metformin reduced oxygen consumption in tumor cells in vitro and in vivo, resulting in reduced TME hypoxia [2]. These results illustrate that metformin promotes CD8 T cells’ proliferation and adaptability in hypoxic TME in different tumor models. Furthermore, metformin activates intracellular AMPK and then directly phosphorylates Ser195 of programmed cell death 1 ligand 1(PD-L1) in the endoplasmic reticulum. Secondly, T cell infiltration into tumor tissues is a prerequisite for anti-tumor efficacy. In triple-negative breast cancer, metformin was found to activate the JNK signaling pathway, increasing the infiltration of functional CD4+ and CD8+ tumor-infiltrating lymphocytes (TILs), and it also suppressed the exhausted phenotype of TILs independently of the JNK pathway, thereby strengthening the anti-tumor immune response [3]. However, most of the studies on metformin for T cells are in CD8+ cells and Th cells, but there is limited concern about memory T cells and other subtypes. These results suggest that metformin plays a crucial role in regulating T cell function to indirectly suppress tumorigenesis or tumor progression (Figure 1A).

B cells and their secreted antibodies play a crucial role in anti-tumor immunity. In ibrutinib-resistant activated B cells, the early growth response factor 1 (EGR1) is upregulated, and its high expression mediates metabolic reprogramming toward oxidative phosphorylation (OXPHOS) via PDP1 transcriptional activation to supply energy to tumor cells. Metformin blocks cellular OXPHOS, thereby disrupting B-cell signal transduction and overcoming ibrutinib resistance in diffuse large B-cell lymphoma [4]. A study on Sjögren’s syndrome demonstrated that metformin controlled B cell differentiation by reducing germinal center B cell populations and serum IgG levels, thereby relieving their immunosuppressive effects [14]. This study uses a relatively low dose of metformin (human equivalent dose of 4 mg/kg/day), which found no significant glucose-lowering effect in the animal model. But its long-term effects remain to be seen. Overall, this is strong evidence that metformin plays an inhibitory role in B cell differentiation (Figure 1B).

Tumor-associated macrophages (TAMs) are a major immune cell population within the TME, and metformin has been proven to regulate TAM polarization through multiple mechanisms. On the one hand, metformin activates the AMPK signaling pathway and inhibits the nuclear factor-kappaB (NF-κB) pathway, thereby reducing pro-inflammatory cytokine release and attenuating inflammation in the TME. It also indirectly promotes TAM polarization toward the M1 phenotype. In breast cancer cells and bone-marrow-derived macrophages, metformin treatment was found to activate the AMPK pathway, inhibiting the release of pro-inflammatory factors such as tumor necrosis factor alpha (TNF-α), IL-1β, and IL-6, and promoting the expression of anti-inflammatory factors like IL-10 [15,16]. On the other hand, metformin can act directly on TAMs to influence their phenotype and function via intracellular signaling regulation. However, transcriptome sequencing revealed that TAM infiltration significantly increased in active lung adenocarcinoma patients with long-term metformin use. Since metformin significantly increased S100A9 expression and secretion of TAMs through the AMPK-CEBP/β pathway [17], it then activates the NF-κB pathway to promote EMT and progression of LUAD. These differences may be due to its poor long-term effects or different tumor types. Beyond simply modulating macrophage polarization, metformin can also recruit a responsive population of macrophages. ScRNA-seq analysis revealed that metformin treatment increases the proportions of B cells, T cells, natural killer T cells, and innate lymphoid cells ILCs in older people ovary, and decreases the proportions of macrophages and dendritic cells. Crucially, within the identified CD45+ immune cell populations, the recruitment of four specific myelomonocytic subsets (macrophages I, II, and III, and monocytes) was found to be significantly enhanced [18]. Together, these findings demonstrate that metformin treatment can remodel the immune landscape in the TME, driven notably by the dynamic alterations in macrophage subpopulations (Figure 1C).

Natural killer (NK) cells are essential components of the innate immune system and play a key role in anti-tumor immunity, and their activation depends on the integration of a series of activating receptors and inhibitory receptors [8]. Metformin regulates p53 via AMPK activation, which in turn amplifies the transcription of NKG2D ligand (NKG2DL) messenger RNA (mRNA), thereby upregulating NKG2D expression to trigger NK cell activation [19]. In a diffuse large B-cell lymphoma PDX model, the combination of metformin with activated NK cells has been shown to be effective in inhibiting the tumor. Furthermore, metformin significantly inhibits c-Myc protein expression by increasing the FOXO3 tumor suppressor levels in ovarian, breast, and hepatocellular tumor cells [20]. Overall, these results indicate that metformin mitigates NK cell inhibition (Figure 1D).

Dendritic cells (DCs) are potent antigen-presenting cells in the immune system, playing a core role in initiating and regulating adaptive immune responses, and targeted delivery of metformin encapsulated in tumor cell-derived exosomes increased pro-inflammatory extracellular ATP (eATP) levels, which enhanced DC maturation and antigen-presenting capacity [21]. A study in ovarian cancer indicated that metformin induces immunogenic cell death (ICD) [22]. Concurrently, following ICD, the expression or release of damage-associated molecular patterns stimulates pattern recognition receptors on macrophages and DCs, leading to antigen-specific CD8+ T cell activation and the initiation of immune responses [23]. However, in autoimmune diseases, activated DCs notably promote effector T cell polarization and exacerbate the disease. Metformin shifts DCs toward a tolerogenic phenotype, thereby reducing surface expression of MHC-II, co-stimulatory molecules, and CCR7; decreasing pro-inflammatory cytokine (TNF-α and IFN-γ) levels; increasing IL-10 levels; upregulating immunoregulatory molecules; and enhancing the capacity to promote Treg differentiation, which together mediate immune tolerance [24]. Ultimately, it could induce tolerance in DCs by reprogramming their metabolic patterns and play anti-inflammatory roles in vitro and in vivo. These distinct responses in tumors and autoimmune diseases are potentially owing to metformin’s context-dependent effects. These results suggest that metformin promotes DCs’ transformation and maturation (Figure 1E).

MDSCs are a population of cells that accumulate under specific pathological conditions such as tumors and chronic inflammation [25]. In the TME, they exert immunosuppressive functions and promote tumor growth by inducing various factors, including IL-6, IFN-γ, IL-1β, TNF-α, vascular endothelial growth factor (VEGF) and GM-CSF [25,26]. Additionally, the CD39/CD73-mediated adenosinergic pathway contributes significantly to MDSC immunosuppression [27]. In ovarian cancer studies, metformin was found to activate the AMPK pathway and inhibit the HIF-1α pathway to suppress CD73/CD39 expression in MDSCs, reducing MDSC-mediated immunosuppression [28], and similar results were observed in a non-small cell lung cancer study [27]. Metformin, acting as a PGE2 antagonist, disrupts MDSC-mediated immunosuppression by reprogramming the PGE2/p50/NO axis, while inhibiting the COX2/PGE2/STAT3 signaling axis regulates the EMT-related molecule expression in the animal model and prostate cancer patients [29]. Hence, these results strongly suggest that metformin is critically important for inhibiting MDSC functions (Figure 1F).

Tumor-associated neutrophils (TANs) exhibit functional polarization or plasticity within the TME. Metformin can lead to reduced AMPK phosphorylation, ultimately decreasing ATP production in neutrophils and inhibiting their function [30]. Specifically, it modulates neutrophil heterogeneity by altering ATP homeostasis and the gut metabolic axis. A metformin-based multifunctional nanocomplex, CS-MET/siTREX1, was constructed as a specific DNA damage amplifier to maximize radio-immunotherapy efficacy and circumvent radiotherapy resistance, and it was found to significantly reduce distal tumor growth by promoting TAN polarization [31]. In general, the above studies confirmed that metformin alters TAN polarization through different molecular mechanisms in various tumors, thus inhibiting tumorigenesis (Figure 1G).

Overall, existing evidence indicates that metformin alters the immune microenvironment by promoting or inhibiting various functions in different immune cells, with the aim of inducing anti-tumor effects, and the effects and mechanisms of metformin in various immune cells are shown in Table 1.

### 2.2. Inflammation Regulation

**Figure 2:**
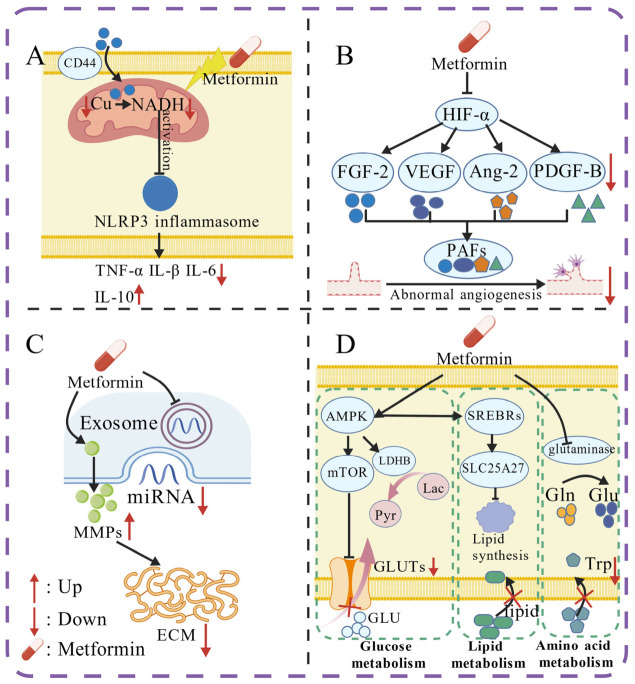
Metformin-mediated reprogramming of tumor microenvironment components. (**A**) Metformin suppresses NLRP3 inflammasome activation and IL-1β/IL-6 secretion while promoting NF-κB inhibition to resolve chronic inflammation. (**B**) Metformin inhibits hypoxia-induced angiogenesis through HIF-1α destabilization and VEGF/FGF-2/PDGF-B downregulation. (**C**) Metformin modulates MMPs expression. (**D**) Metformin reprograms metabolic pathways: inhibiting glycolysis, reducing lipid uptake and altering amino acid utilization.

Nonresolving inflammation within the TME is a key contributor to tumor proliferation, migration, and invasion, as well as therapeutic resistance [38]. In mouse bone-marrow-derived macrophages cultivated with metformin (0.5 mM), it is able to inhibit NLRP3 inflammasome activation and IL-1β production, as well as inflammasome-independent IL-6 secretion, through various pathways such as JNK, complex I and ox-mtDNA inhibition. It ultimately relieves the excessive inflammatory response in the acute respiratory distress syndrome animal model. However, it is still unclear whether short-term metformin treatment fully mimics long-term drug treatment. Additionally, it blocks the ATP-dependent synthesis of LPS-induced mtDNA, an NLRP3 ligand, thereby alleviating inflammatory responses [39]. However, in actual clinical studies, metformin concentration and targetability in tumor tissues were not high. A study targeting the chemically reactive mitochondrial copper pool revealed that this pool catalyzes NAD(H) redox cycling by activating H_2_O_2_, and NAD+ maintenance induces inflammation [36]. Solier et al. utilized metformin dimers to target mitochondrial copper (II), which induced a reduction in the NAD(H) pool and then inhibited macrophage activation and dampened the inflammatory response. Furthermore, in preeclamptic rats [40], metformin treatment effectively inhibited the elevation of pro-inflammatory serum markers TNF-α and IL-6, thereby reducing L-NAME-induced inflammation. Together, these results indicate that metformin exerts anti-tumor effects by inhibiting the release of pro-inflammatory factors and activating anti-inflammatory pathways (Figure 2A).

### 2.3. Vessel Regulation

Tumor growth and metastasis are heavily reliant on neovascularization. However, the tumor vasculature is typically structurally abnormal and functionally disordered. It is characterized by incomplete vessel walls, hyperpermeability, and disorganized vascular networks. As previously reported, pro-angiogenic factors such as vascular endothelial growth factor (VEGF), fibroblast growth factor 2, angiopoietin-2 and platelet-derived growth factor subunit B are frequently overexpressed in malignancies like breast cancer [41]. However, metformin administration significantly inhibits excessive angiogenesis in the murine breast cancer model. Specifically, it inhibits the expression of these factors through the direct HIF-α downregulation, leading to angiogenesis suppression. Additionally, it was found that high PDGF-B expression was detected in the metastatic breast cancer model. By reducing tumoral PDGF-B, the metformin (100 μM) treatment resulted in angiogenesis suppression and a more mature vasculature of metastatic breast cancers, thus curbing the distant metastasis and enhancing the efficiency of drug delivery. As the structure determines the function, improved vascular maturity led to an increase in the blood perfusion of tumors, thus allowing more chemo-drugs or therapeutic particles to be delivered into the tumors [11]. Furthermore, Tokumasu et al. discovered that the combination of metformin and anti-PD-1 therapy promotes pericyte coverage of tumor endothelial cells to improve blood perfusion and mitigates vascular hyperpermeability by upregulating VE-cadherin expression [42]. In summary, these results suggest that metformin may inhibit tumor invasion and metastasis by inhibiting abnormal angiogenesis and may be more effective when combined with immunotherapy (Figure 2B).

### 2.4. Matrix Regulation

The ECM of the TME constitutes a complex network composed of collagen, laminin, fibronectin, hyaluronic acid and proteoglycans, and aberrant collagen deposition within the ECM serves as a primary component of this dense stromal barrier. However, matrix metalloproteinases (MMPs), a class of zinc-dependent endopeptidases, are capable of degrading various ECM components, including collagen and fibronectin [43]. Targeted delivery of metformin to M2-like TAMs has been shown to promote MMP expression in macrophages, inducing tumor ECM degradation and facilitating CD8+ T cell infiltration [44]. Additionally, three different doses of metformin (5, 10, and 25 mM) treatment with primary breast cancer cells downregulated MMP-9 expression without affecting MMP-2 and suppressed NF-κB activity in a dose-dependent manner, thereby inhibiting tumor invasion and metastasis [16]. However, we have to admit that the high concentration limits its clinical use.

Exosomes are extracellular vesicles released by most cell types, including cancer cells. Specifically, tumor-derived exosomes contain a variety of molecules, such as microRNAs (miRNAs) and proteins [45]. MiRNAs, including miR-21, miR-155, and miR-182, are known to promote tumorigenic angiogenesis, oncogenesis [46] and chemoresistance [47]. Importantly, metformin has been found to inhibit the expression and exosomal loading of these miRNAs, thereby suppressing glioblastoma malignant progression [48]. In general, metformin inhibits malignant tumor progression by modulating the expression of related stromal molecules (Figure 2C).

### 2.5. Metabolic Reprogramming

Glycolysis serves as the predominant energy metabolic pathway for both tumor cells and CAFs, and the resulting lactate accumulation is the primary driver of microenvironmental acidification. Lactate accumulation facilitates multiple behaviors such as immune escape and signal transduction modulation [49]. Metformin inhibits glycolysis through multifaceted mechanisms. Beyond the well-established AMPK-mTOR and mitogen-activated protein kinases/extracellular signal-regulated kinase (MAPK/ERK) pathways [9,10], metformin has been found to regulate the expression and activity of key glycolytic enzymes. For instance, in primary hepatocellular carcinoma, metformin inhibits the expression of hexokinase 2 and the pyruvate kinase isoform M2, both of which are critical for the rapid glycolytic flux of tumor cells [50]. Furthermore, Benjamin et al. discovered that metformin promotes lactate dehydrogenase B expression, which is vital for converting lactate into pyruvate, thereby lowering lactate levels [51]. Furthermore, metformin substantially decreases the accumulation of extracellular lactic acid, thereby reversing TAMs toward an immunosuppressive phenotype and inhibiting CAFs activation. Additionally, it also prevents VEGF-driven aberrant angiogenesis, restores VE-cadherin-mediated endothelial junctions, and enhances pericyte coverage to achieve vascular normalization. Further studies suggest that metformin may limit the glycolytic substrate supply by modulating glucose transporter (GLUT) expression to inhibit glucose uptake [52]. In conclusion, research on the molecular mechanisms of glycometabolism is closely related to metformin-induced tumor cell proliferation and tumorigenesis.

Abnormal lipid metabolism within the TME is another significant driver of tumor progression. Tumor cells and stromal cells release free fatty acids (FFAs) and lipid peroxides, leading to elevated FFA levels in the TME. Excessive FFAs not only serve as an energy source to directly fuel tumor growth and proliferation but also indirectly promote progression by eliciting inflammatory responses and inhibiting immune cell function [53]. Metformin exerts anti-tumor effects by inhibiting lipogenesis, promoting fatty acid oxidation, reducing lipid uptake, and decreasing ROS generation [54], thereby alleviating lipotoxicity, suppressing inflammation and enhancing immune cell function. Especially, it can disrupt the fatty acid oxidation required for MDSC-mediated T cell suppression. SLC25A47, a hepatic NAD+ transporter regulating lipogenesis via the SIRT3-AMPKα-SREBP pathway, is transcriptionally activated by metformin to inhibit lipid synthesis and delay tumor progression [55], suggesting that metformin plays an essential role in inhibiting lipid synthesis.

Amino acid metabolism also plays a pivotal role in TME metabolic reprogramming. Metformin remodels the TME to exert anti-tumor effects by regulating the metabolism of key amino acids. Specifically, metformin has been reported to inhibit glutamine metabolism in malignancies such as osteosarcoma, hepatocellular carcinoma, and pancreatic cancer [56,57,58] by reducing glutaminase activity, thereby depriving tumor cells of essential energy and carbon sources. Furthermore, metformin remodels tryptophan metabolic partitioning. Restricting tryptophan uptake in colorectal cancer cells, it restores tryptophan utilization by CD8+ T cells [12]. Additionally, metformin alleviates tryptophan depletion induced by excessive consumption by tumor and immune cells in the TME and promotes the degradation of tryptophan metabolites such as kynurenine [59], thereby relieving T and NK cells suppression and potentiating anti-tumor immune responses.

In summary, these studies illustrate the function of metformin in inflammation regulation, vascular regulation, matrix regulation, metabolic reprogramming, and its molecular mechanism in the TME (Figure 2D).

## 3. Metformin Mediates the Crosstalk Between TME Components to Suppress Tumor Progression

**Figure 3:**
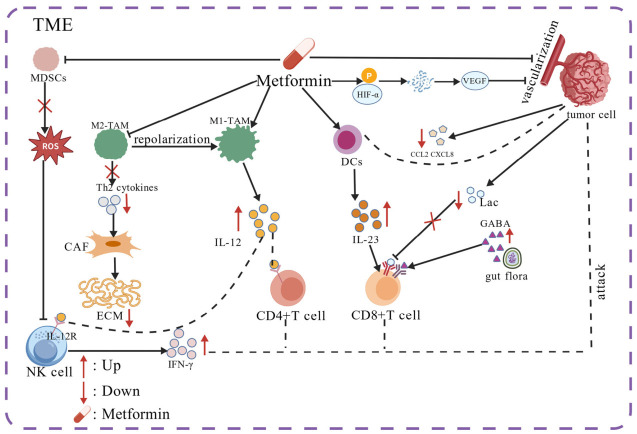
Metformin crucially orchestrates complex interactions between these components, thereby profoundly influencing tumor progression.

The TME is not merely an accumulation of various components but rather a dynamic and highly interactive complex ecosystem. Distinct components interact through diverse mechanisms such as direct contact, paracrine signaling and exosome-mediated communication, and these interactions collectively shape the characteristics of the TME and ultimately drive tumor growth, invasion, and metastasis, as well as therapeutic responses. Beyond acting directly on specific cell types or molecules within the TME, metformin is capable of mediating and regulating the complex crosstalk among these diverse components, thereby inhibiting malignant progression (Figure 3).

### 3.1. Metformin in Immunity and Inflammation Regulation

Immunosuppression and chronic inflammation within the TME are pivotal drivers of tumor progression. Metformin regulates immune cell functions and inflammatory cytokine networks via multiple targets, thereby disrupting immune tolerance and suppressing inflammatory responses to remodel anti-tumor immunity.

IL-12 and IL-23 are heterodimeric cytokines produced by antigen-presenting cells that regulate lymphocyte activation and differentiation, sharing IL-12Rβ1 as a receptor signaling subunit [60]. IL-12 signals through the receptor complex of IL-12Rβ1 and IL-12Rβ2, which are expressed on NK and T cells, activating the JAK-STAT4 pathway to promote immune interferon (IFN) γ expression and the polarization of CD4+ T cells toward a Th1 phenotype, thereby enhancing NK cell cytotoxicity [60,61]. Metformin, via AMPK activation, promotes the release of factors such as IL-12 and IL-23 from M1-like macrophages and DCs, thereby indirectly enhancing NK cell function [62]. As mentioned above, metformin promotes the release of IL-12 in the TME to enhance immune function.

MDSCs suppress T cell activity by secreting factors such as IL-10 and TGF-β, which act as critical accomplices in tumor immune escape [63]. Metformin reduces CXCL1 expression via the AMPK-DACH1-CXCL1 axis, hinders the migration and accumulation of MDSCs and attenuates their suppressive function [64]. Some activated MDSCs produce ROS and inducible nitric oxide synthase, leading to a reversible decline in CTL activity and IFN-γ production or even dismantling effector T cell responses. However, metformin can reverse these effects.

The M2 phenotype of TAMs is closely associated with tumor progression, but metformin reverses this polarization through multiple pathways [15,16]. Metformin treatment leads to AMPK upregulation, which modulates p65 phosphorylation and reduces the secretion of Th2 cytokines (including IL-4, IL-10 and IL-13), resulting in the reprogramming of TAMs toward M1 macrophages [65]. In short, metformin reduces the release of tumor-promoting factors, leading to the inhibition of macrophage polarization into TAMs.

Chemokines such as CCL2 and CXCL8, which are secreted by tumor cells, recruit pro-inflammatory immune cells like neutrophils and monocytes, establishing a pro-metastatic microenvironment [64]. Following metformin-induced AMPK activation, the p85α regulatory subunit of PI3K is directly phosphorylated, inhibiting AKT/mTORC1 signaling [66]. This reduces NF-κB translocation into the nucleus and activates protein-1, downregulating CCL2 and CXCL8 expression and thereby suppressing the expression of these chemokines. Therefore, metformin plays a crucial role in regulating the expression of chemokines, which has important implications for immunity.

### 3.2. Metformin in Vascular and Matrix Regulation

Tumor growth and metastasis heavily rely on a dysfunctional tumor vasculature and a dynamically remodeled ECM [67].VEGF is a key factor widely reported to promote angiogenesis [11,41,67]. Weon-Kyoo You et al. demonstrated in multiple experimental studies that reduced binding of VEGF-A to a defective vascular ECM leads to impaired tumor vascularization, thereby hindering tumor growth [68]. Furthermore, reduced collagen, laminin, and fibronectin deposition, along with the loss of integrin-binding sites in the basement membrane, exerts additional negative effects on vascular cell adhesion, growth, and survival [69]. Therefore, regulating VEGF expression represents a crucial target for inhibiting tumor growth. However, elevated HIF-1α expression under hypoxic conditions within the TME is associated with increased VEGF-A expression [68,70,71]. As a classic AMPK agonist, metformin can directly phosphorylate HIF-1α, thereby decreasing its stability and inhibiting its transcriptional activity. Additionally, factors such as TGF-β, PDGF and IL-6 stimulate quiescent CAF to activate it [67]. Metformin combination with liraglutide on HUVEC indicated a substantial increase in VEGF, PDGF, and MMP-9, thus inhibiting its migration [72]. Furthermore, PDGF upregulated the expression of α-SMA, as well as type I collagen and fibronectin, in HSCs, while these protein levels were decreased when treated with metformin [73]. These results indicate that metformin indirectly and directly downregulates PDGF expression to reduce ECM secretion. A study on pancreatic ductal adenocarcinoma revealed that combination therapy involving oxaliplatin, photodynamic therapy and metformin suppressed CAF function in MIA PaCa-2 microtumors, thereby overcoming CAF-induced therapeutic resistance [74]. However, in this 3D microtumor model cultured PDAC lines and CAFs, it still differs from the actual human environment because of immune, inflammatory and other components. Generally, metformin disrupts vascular–stromal regulatory mechanisms and suppresses angiogenesis, thereby exerting anti-tumor effects.

### 3.3. Metformin in Metabolic and Immune Reprogramming

Tumor cells typically exhibit a metabolic profile characterized by high rates of glycolysis, converting glucose to lactate even under oxygen-sufficient conditions (the Warburg effect) [75]. The massive accumulation of lactate leads to TME acidification, which inhibits the function of various immune cells and promotes nonresolving inflammation [76], including the suppression of immune cell proliferation [77], cytotoxicity [78] and effector molecule secretion [76,77,78]. Furthermore, lactate promotes histone lysine lactylation, leading to transcriptional regulation. Increasing evidence suggests that lactate itself can function as a signaling molecule, exerting regulatory effects by binding to specific surface receptors on immune cells, such as G-protein-coupled receptor 81 [79], or by being transported into cells via monocarboxylate transporters [51,80]. The combined use of syrosingopine and metformin [51] can induce a loss of NAD+ regeneration capacity, thereby blocking glycolysis and reducing lactate production in cancer cells. Concurrently, this combination inhibits MCT1 and MCT4 activity to reduce lactate efflux, thereby reducing the lactate distribution within the TME. This ultimately alleviates lactate-mediated suppression of T cell and NK cell immunosurveillance [77] and mitigates the promotion of PD-1 expression on regulatory T cells [78]. These results suggest that metformin affects the function of immune cells by regulating lactate levels.

OXPHOS, another critical energy production pathway in tumor cells, is also abnormally enhanced, accompanied by increased generation of ROS. Excessive ROS production often leads to oxidative stress, which subsequently damages DNA, proteins, and lipids; promotes inflammatory responses; and creates favorable conditions for tumor initiation and progression. These molecules can promote the release of pro-inflammatory factors such as IL-6 and TNF-α, as well as PD-L1 expression [81,82], thereby modulating anti-tumor immunity and inflammatory responses. Metformin treatment has been found to significantly inhibit methylglyoxal-stimulated ROS production. It enhances signaling via the ROS-mediated PI3K/Akt and Nrf2/HO-1 pathways and significantly elevates downstream antioxidant levels [83]. Simultaneously, as a mitochondrial complex I inhibitor, metformin prevents TNF-induced mitochondrial ROS production in human macrophages [84]. Therefore, metformin can inhibit oxidative damage, apoptosis, and inflammation via OXPHOS regulation.

### 3.4. Metformin in Metabolic and Gut Microbiota Reprogramming

In recent years, the importance of gut microbiota and their metabolites in regulating systemic immune responses and tumor immunotherapy efficacy has become increasingly prominent [85]. Metformin has been reported to alter the composition and function of the gut microbiota, promote the growth of beneficial bacteria and influence the production of microbial metabolites [86]. Among these, short-chain fatty acids such as butyrate possess various immunomodulatory functions, including inducing cytotoxic granzyme B, IFN-γ and TNF-α production [87], as well as enhancing CD8+ T cell cytotoxicity [88]. Mechanistically, butyrate can directly bind to toll-like receptor 5 on CD8+ T cells to induce their activity via NF-κB signaling activation [87] or enhance the killing function of CD8+ T cells against gastric cancer cells via the GPR109A/HOPX axis [89]. Through 16S rRNA and metagenomic sequencing, Wang et al. discovered that metformin induces an increase in γ-aminobutyric acid (GABA)-producing bacteria in the gut; this is attributed to elevated levels of key GABA synthesis enzymes—such as glutamate decarboxylase and putrescine aminotransferase [90]. In summary, metformin is able to alter the gut microbiota and its metabolites to mediate anti-tumor immune responses in the TME.

## 4. Molecular Mechanisms Underlying the Anti-Tumor Effects of Metformin in the TME

Metformin, widely recognized as a broad-spectrum anti-diabetic medication, has recently been identified to possess significant anti-tumor potential [91]. Its anti-tumor efficacy is not only confined to the direct inhibition of tumor cells. Crucially, it exerts therapeutic effects by regulating the complex TME [19,22,35,92]. Metformin-mediated intervention in the TME indirectly inhibits tumor cells via various pathways or directly impacts tumor cells, primarily involving the canonical AMPK signaling pathway [10,15,16], as well as AMPK-independent signaling pathways including PI3K/Akt/mTOR [66], JNK [3], HIF-1α [93] and STAT3, which are elaborated below (Table 2).

### 4.1. AMPK Signaling Pathway

The AMPK trimer is a critical regulator of cellular energy metabolism; it is composed of a catalytic α subunit and two regulatory β and γ subunits, each possessing distinct isoforms [106]. This heterotrimeric structure coordinates intricate energy-regulating functions, with the β and γ subunits playing roles in maintaining complex stability and binding AMP as a stimulator, respectively [107].

**Figure 4:**
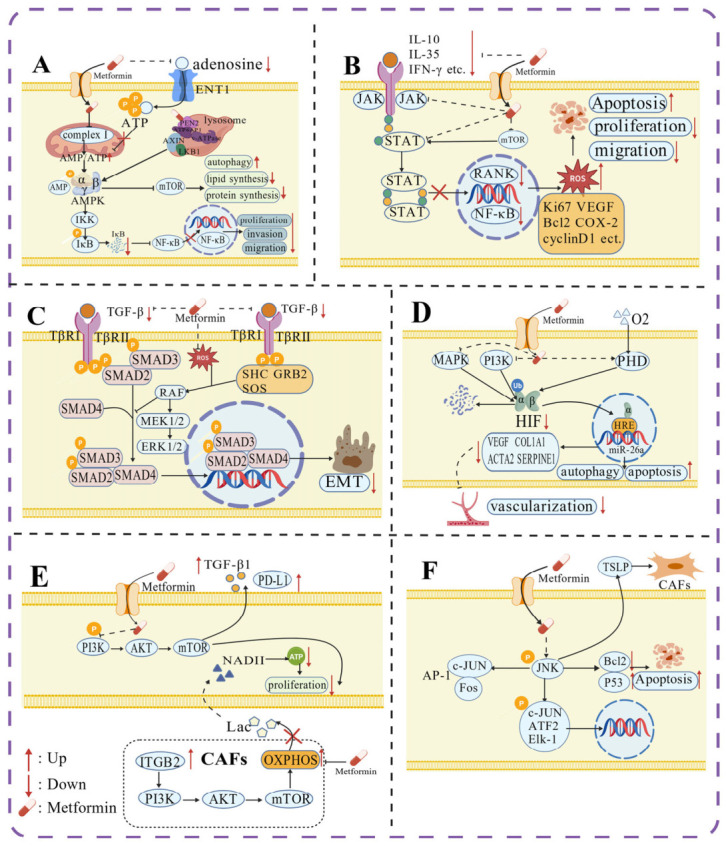
Molecular mechanisms of metformin-mediated TME reprogramming. (**A**) Metformin acts as an AMPK agonist, orchestrating TME remodeling to inhibit tumor progression. (**B**) STAT3 signaling is regulated by metformin through multiple mechanisms. (**C**) TGF-β regulates context-dependent transcription through SMAD and non-SMAD pathways and exert their anti-tumor effects. (**D**) HIF-1α expression and activity are suppressed by synthesis inhibition, degradation promotion and activation blockade, culminating in reduced collagen deposition, diminished adhesion stability and anti-angiogenic effects. (**E**) Metformin impairs the energy supply from CAFs to the tumor by inhibiting the PI3K/AKT/mTOR signaling pathway. (**F**) Metformin mediates apoptosis and inflammatory response by regulating the JNK signaling pathway.

Metformin can indirectly regulate tumor cells in the TME by inhibiting adenosine release from MDSCs. This adenosine is otherwise taken up by tumor cells by ENT1 and sequentially phosphorylated to ATP, thereby regulating cellular ATP and adenosine levels [108]. Additionally, metformin directly modulates AMPK activation in various tumor cells such as HEK293, MC3T3-E1, RAW264.7, MEFs, BCPAP and BHP10-3SC cells. But the treatment with these cell lines (HEK293: 5 mM or 0.2 mM, MC3T3-E1 and RAW264.7: 3 mM, MEFs: 0.2 mM, BCPAP and BHP10-3SC: 20 mM) has a different dosage to achieve the best effect. This difference may be due to different activation pathways or different uptake efficiencies in different cell lines. By inhibiting mitochondrial complex I, it reduces ATP production and induces energy stress with high dosage, leading to elevated AMP levels that activate AMPK [109]. Alternatively, it utilizes the lysosomal proton pump v-ATPase to regulate the v-ATPase-AXIN-AMPK axis with low dosage, thereby activating AMPK without altering AMP or ADP levels [10,110]. For instance, metformin binds directly to PEN2 on lysosomes, promoting the interaction between PEN2 and the ATP6AP1 subunit of v-ATPase, thereby intersecting with the lysosomal v-ATPase-AXIN-AMPK axis [10]. Metformin activates the AMPK signaling pathway through its direct interaction with lysosomal PEN2 or mitochondrial complex I inhibition. Additionally, metformin-induced AMPK activation phosphorylates and inhibits downstream mTOR activity, thereby suppressing anabolic processes such as protein and lipid biosynthesis while enhancing catabolic processes like fatty acid oxidation [111]. Furthermore, AMPK activation blocks the nuclear translocation of NF-κB p65 in activated macrophages within inflamed skin tissues, preventing its transcriptional function and inhibiting IL-1β secretion [112]. Further research indicates that AMPK activation inhibits IκB kinase (IKK) activity. Upon phosphorylation, IκB undergoes ubiquitin-mediated degradation, releasing NF-κB to translocate it into the nucleus and execute its transcriptional functions. Consequently, inhibiting IKK stabilizes IκB, thereby preventing the nuclear translocation and activation of NF-κB [113]. Ultimately, metformin inhibits different tumor progression in multiple tumor types via the AMPK/mTOR pathway, including proliferation in esophageal squamous cell carcinoma cells [94], proliferation, invasion, and migration in colorectal cancer cells [95], and tumor growth in these animal models. In general, these results indicate that metformin can induce AMPK activation by accommodating the adenosine level in the TME or specifically affect the tumor cells, leading to impaired metabolism in tumor cells (Figure 4A).

### 4.2. STAT3 Signaling Pathway

The STAT proteins constitute a family of cytoplasmic transcription factors, primarily comprising STAT1, STAT2, STAT3, STAT4, STAT5a, STAT5b, and STAT6, which mediate diverse intracellular signaling cascades. Among them, STAT3 is highly expressed in the majority of malignancies, participating in biological processes such as cell proliferation, survival, differentiation and angiogenesis to drive tumor progression [114].

Cytokines within the TME exert multifaceted regulatory effects on tumor cells, with the JAK/STAT signaling pathway being paramount [115]. Receptor dimerization induces JAK phosphorylation, followed by the phosphorylation of cytokine receptors, providing docking sites for SH2 domain-containing STATs. Upon recruitment to cytokine receptors, STATs undergo phosphorylation, dimerization and translocation to the nucleus [116]. However, metformin may directly inhibit STAT3 phosphorylation and its nuclear translocation, thereby suppressing its function. Additionally, metformin can indirectly regulate STAT3 signaling by adjusting other factors such as SIRT1, PTPRD, and mTOR [117]. Consequently, its treatment (1 mM) resulted in strong and additive proliferation and migration inhibition in glioblastoma cells [96]. A common phenomenon is the hyperactivation of the JAK/STAT3 pathway via IL-6 and IL-6R overexpression. Phosphorylated STAT3, in turn, binds to the IL-6 promoter, inducing the release of immunosuppressive factors such as IL-6, IL-10, TGF-β and VEGF, thereby establishing a STAT3 positive feedback amplification loop [118]. Metformin reduces the expression of STAT3 stimulators like IL-6, indirectly mediating the inhibition of STAT3 activation [39]. In glioblastoma research, it was found that following nuclear translocation, STAT targets the transcriptional activity of oncogenes (including ROS, RANK, Egr-1, c-Myc, Bcl-XL, NF-κB, AP-1 and p53). Furthermore, metformin can also act directly on glioblastoma cells, reducing their STAT3 phosphorylation levels and thereby inhibiting proliferation and migration. Nevertheless, whether STAT3 is a direct target remains unknown, and how it indirectly inhibits this signaling pathway through other mechanisms requires further elucidation. Subsequently, metformin downregulates IL-6 expression within the TME, inhibiting the JAK/STAT3 pathway and consequently suppressing cell proliferation, migration, and invasion (Figure 4B).

### 4.3. TGF-β Signaling Pathway

TGF-β serves as the prototype of the TGF-β superfamily, which comprises TGF-β, activins, nodals, bone morphogenetic proteins, growth and differentiation factors and other related factors [119]. It is primarily secreted and sequestered within the ECM as a latent complex [120], and its biological functions are exerted only when activated TGF-β binds to the TGF-β receptor complex. Consequently, TGF-β activation is critical for its function [121].

Metformin reduces TGF-β release from immune cells such as B and T cells [34]. This reduction impedes the stimulation of distinct downstream signaling pathways (SMAD and non-SMAD pathways), thereby regulating TGF-β context-dependent transcription to exert anti-tumor effects. The SMAD pathway represents the canonical route wherein TGF-β is recognized by TβR II, which possesses an intracellular kinase domain. TβR II then recruits and phosphorylates TβR I at the glycine/serine-rich “GS domain” via its serine/threonine kinase activity. Subsequently, the activated TβR I phosphorylates receptor-regulated SMAD (R-SMAD) proteins and facilitates the binding of the R-SMAD complex with Co-SMAD/SMAD4 to form a trimeric complex. This trimeric complex translocates into the nucleus and accumulates to regulate target gene expression [122]. Collectively, pathways and downstream cascades activated by TGF-β via phosphorylation, acetylation, SUMOylation, ubiquitination, and protein–protein interactions are termed non-SMAD signaling pathways [123]. Treatment with metformin (10 mM) in the pancreatic cancer cells suppresses morphological changes, EMT and migration. Additionally, in a mouse metastatic tumor model treated with metformin at 125 mg/kg (equivalent to 600 mg/average size individual of 60 kg in humans), liver metastasis was reduced, but tumor growth in situ was not significantly different [97]. The dose difference between in vivo and in vitro may be due to the TME in vivo. Among these, metformin reduces TGF-β expression in the TME, then indirectly affects the SAMD signaling pathway in the tumor cells, ultimately promotes the EMT in the TME-dependent manner and facilitates fibrosis in cancer metastasis (Figure 4C).

### 4.4. HIF-1α Signaling Pathway

HIF-1 is a heterodimeric protein composed of two subunits: HIF-1α and HIF-1β. HIF-1α functions as a critical transcription factor that is stabilized and activated under hypoxic conditions [124]. Additionally, activated HIF-1α dimerizes with HIF-1β and binds to specific hypoxia response elements within the promoters and enhancers of effector genes, thereby promoting the expression of several genes, most notably pro-angiogenic factors such as VEGF.

Metformin can downregulate HIF-1α expression levels by regulating its synthesis, degradation, and activation. It also affects the activity of PHD enzymes and factors inhibiting HIF or the interaction between VHL and HIF-1α, thereby promoting the ubiquitin-mediated degradation of HIF-1α or suppressing its stability [125]. HSP90 binding to HIF-1α stimulates its activation via two mechanisms: firstly, it prevents HIF-1α degradation through a VHL-independent proteasome pathway. Secondly, it maintains the HIF-1α protein in active conformation, facilitating p300 recruitment and transactivation initiation [98]. Additionally, HIF-1α expression can be influenced by regulating its synthesis processes, such as the activation of the PI3K and MAPK pathways [124]. Metformin can also directly or indirectly inhibit the activation of the PI3K and MAPK signaling pathways [126], thereby inhibiting HIF-1α synthesis. Collectively, these findings indicate that metformin indirectly reduces HIF-1α expression in both tumor cells and the TME via these pathways.

In studies on liver fibrosis induced by congestive hepatopathy, metformin was found to ameliorate liver fibrosis by reducing collagen, fibronectin, α-smooth muscle action and HIF-1α expression. The result is also attributed to the inhibition of HSC activation and the mTOR/HIF-1α signaling pathway [99]. Additionally, the metformin-induced reduction in HIF-1α levels alleviates collagen deposition within adhesive tissues mediated by several HIF-1α target genes (specifically SERPINE1, ACTA2, and COL1A1) [127]. This effect arises because metformin-mediated HIF-1 inhibition reduces myofibroblast activation and EMT, ultimately leading to decreased collagen deposition and diminished adhesion stability. Under hypoxic conditions, HIF-1α directly binds to the promoter region of endothelial DGKG to activate its transcription. Additionally, upregulated DGKG promotes ZEB2 deubiquitination by recruiting ubiquitin-specific peptidase 16, which increases TGF-β1 secretion, thereby inducing tumor angiogenesis and Treg differentiation [128]. Beyond indirectly influencing tumor development via TME modulation, metformin-mediated downregulation of HIF-1α expression can also act directly on tumor cells. A combination of metformin, sodium oxamate and doxorubicin in colorectal cancer cells downregulates HIF-1α. Its downregulation inhibits miR-26a expression and ultimately mediates autophagy and apoptosis [5]. These results indicate that metformin can decrease the HIF-1α levels in various pathways, which results in a reduction in collagen deposition and adhesion stability and ultimately inhibits EMT and tumor progression (Figure 4D).

### 4.5. PI3K/Akt/mTOR Signaling Pathway

The PI3K/Akt/mTOR signaling pathway constitutes a pivotal intracellular signaling network in eukaryotic cells, playing central roles in regulating cell growth, proliferation, survival, protein synthesis, metabolism, angiogenesis, and resistance to apoptosis.

Activated PI3K/AKT/mTOR signaling enhances glycolysis of CAFs, leading to increased lactate release. This lactate is subsequently taken up by tumor cells to generate NADH for ATP synthesis, ultimately promoting tumor cell proliferation. Metformin intervention disrupts OXPHOS in CAFs, thereby severing this metabolic energy supply to tumor cells [129]. Furthermore, metformin can directly phosphorylate the PI3K regulatory subunit p85α via AMPK activation, thereby inhibiting AKT/mTORC1 signaling [66]. Additionally, metformin mediates PI3K inhibition by reducing the phosphorylation of insulin receptors [101]. The PI3K signaling pathway and glycolysis form a positive feedback loop: PI3K activation drives glycolytic metabolic reprogramming, while targeted inhibition of glycolysis triggers an energy crisis that reversely blocks PI3K signaling [130]. The classical mechanism of metformin’s anti-diabetic effect involves regulating the intracellular glycolysis pathway. A study on gastric cancer revealed that metformin inhibits glycolysis in tumor cells, thereby reducing ATP production and inhibiting the downstream PI3K/AKT/mTOR signaling pathway, which ultimately inhibits tumor stemness, EMT, and metastasis [102]. These findings illustrate that while exerting its anti-diabetic functions, metformin simultaneously achieves anti-tumor effects through crosstalking glycolysis with these signaling pathways. In short, metformin has multiple mechanisms for indirectly inhibiting the activation of PI3K/AKT/mTOR signaling (Figure 4E).

### 4.6. JNK Signaling Pathway

JNK, also referred to as a stress-activated protein kinase, is a pivotal member of the MAPK family. Its activation is mediated by a sequential phosphorylation cascade involving upstream MAP3Ks (such as MEKK1/2/3 and ASK1) and MAP2Ks (such as MKK4/7) [131]. Metformin activates JNK via the MAPK signaling cascade.

In triple-negative breast cancer, metformin has been shown to increase JNK phosphorylation while decreasing RSK2 and CREB phosphorylation, thereby enhancing the infiltration of functional CD4+ and CD8+ TILs. Additionally, metformin promotes glutamine deprivation and activates the ASK1-JNK pathway to alleviate polycystic ovary syndrome [103]. Activated JNK phosphorylates a variety of substrates, including transcription factors such as c-Jun, ATF2 and Elk-1, thereby regulating gene expression. Furthermore, JNK regulates mitochondrial apoptosis-related proteins of the Bcl-2 family (Bcl-2, Bcl-xl, Bad, Bim, and Bax) as well as the tumor suppressor p53, thereby mediating the pro-apoptotic functions of JNK [132,133]. However, JNK can also enhance the function of CAFs by upregulating the expression of thymic stromal lymphopoietin in bladder cancer. This suggests that metformin plays an essential role in regulating JNK activation in the TME and the tumor cells, thereby attenuating CD8+ T cell cytotoxicity and promoting tumor cell apoptosis (Figure 4F).

### 4.7. Other Signaling Pathways

Beyond these classical signaling pathways, metformin can also regulate other signaling pathways. Mitochondrial mGPDH, the key enzyme connecting OXPHOS and glycolysis, is a target of the anti-diabetic drug metformin in the liver. It has also been found to directly target mGPDH, which acts by shifting the NADH/NAD+ ratio to inhibit OXPHOS and ATP synthesis, thereby inducing metabolic alterations in thyroid cancer cells and suppressing proliferation [104]. Additionally, high mobility group box 1 (HMGB1) is released by necrotic cells and induces inflammatory responses via its cytokine-like activity. A study on TNBC revealed that metformin directly binds to free HMGB1 in the TME [105]. This interaction competitively inhibits HMGB1 from binding to the RAGE receptor on MDA-MB-231 cell membranes, thereby blocking downstream NF-κB signaling, ultimately suppressing the EMT. Generally, these results indicate that metformin can suppress tumor cell proliferation and the EMT either by directly targeting intracellular mGPDH or by competitively binding to HMGB1 in the TME.

### 4.8. Interactions Among Metformin-Regulated Signaling Pathways

Rather than modulating isolated signaling pathways, accumulating evidence indicates that metformin functions as a multi-target orchestrator, initiating a highly interconnected regulatory network. Specifically, the AMPK pathway serves as the core upstream driver; upon activation, AMPK cross-regulates mTORC1 activity, which in turn forms a negative feedback loop with the PI3K/AKT axis. Furthermore, apart from directly mitigating HIF-1α activation, metformin coordinates an intricate crosstalk with the AMPK signaling cascade. Specifically, metformin-activated AMPK directly constrains the Warburg effect by suppressing HIF-1α transcriptional activity, while concurrently limiting nonresolving inflammation via the direct phosphorylation of NF-κB subunits, which strictly restricts their nuclear translocation [134]. Additionally, among PI3K/Akt/mTOR and NF-κB pathways regulated by metformin, PI3K-mediated Akt phosphorylation prompts mTORC1 activation, which subsequently initiates a precise negative feedback loop that attenuates PI3K/Akt signaling, thereby preventing NF-κB hyperactivation and maintaining microenvironmental inflammatory homeostasis [135]. From our perspective, these multi-targets synergy underscore metformin’s primary therapeutic superiority over traditional specific small-molecule inhibitors. While targeted therapies frequently trigger clinical resistance through compensatory pathway activation, metformin avoids this problem by resetting the entire tumor network crossing these pathways. Consequently, the anti-tumor efficacy of metformin is not derived from the potency of a single isolated target, but rather from the harmonious convergence of these interactive signaling pathways.

**Figure 5:**
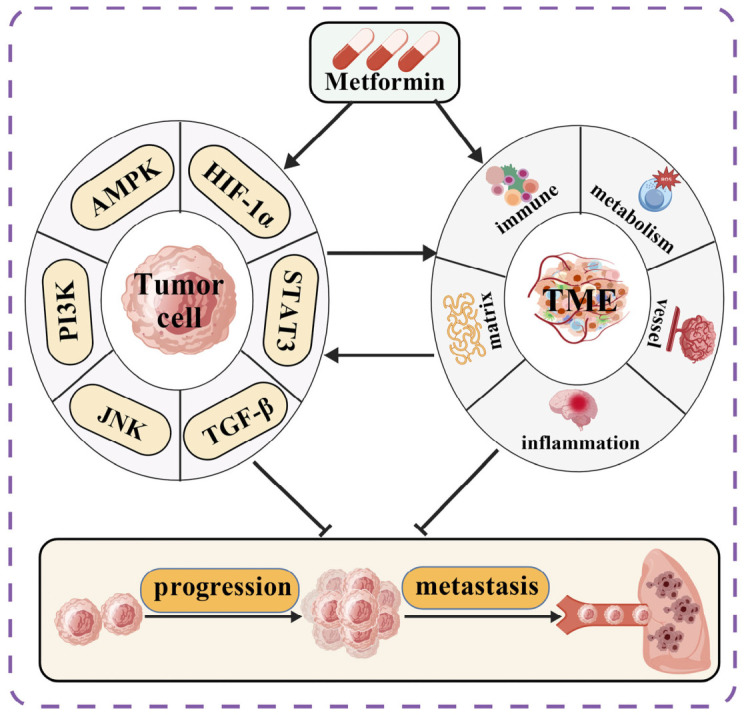
Metformin orchestrates crosstalk between immune remodeling, vascular normalization, stromal modulation, inflammatory reprogramming and metabolic alteration within the TME, and then regulates distinct signaling pathways and their interaction, collectively impacting tumor progression and metastasis.

In summary, metformin may act directly on cancer cells to inhibit signaling through key oncogenic pathways, including AMPK, mTOR, mGPDH, and PI3K/AKT, leading to decreased cell proliferation. On the other hand, it can also modulate components of the TME changes by regulating tumor cells that form a positive feedback loop to further impact the tumor cells through HIF-1α and HMGB1 pathways. Ultimately, these combined mechanisms effectively inhibit tumor progression and metastasis. Taken together, metformin achieves its anti-tumor effects by modulating tumor cells, the TME, and the crosstalk between them (Figure 5).

## 5. Clinical Potentials of Metformin in Cancers

### 5.1. Pharmacokinetics and Therapeutic Potential of Metformin in Cancers

The vast dosage discrepancy of metformin between in vitro and in vivo research in various tumor types is likely linked to distinct differences in its bioavailability, tissue distribution, mitochondrial accumulation, tumor penetration and microenvironment. In vitro experiments habitually rely on high concentrations (mM), ranging from 5 mM to 25 mM, to force energy stress via mitochondrial complex I inhibition. In contrast, regular clinical management for type 2 diabetes yields a steady-state plasma concentration of only around 30 µM [136]. However, a standard clinical daily dose of up to 2000 mg (approximately 15 mmol) can achieve significantly higher localized tissue distributions, such as reaching 140 µM in the liver [137]. Furthermore, in colorectal cancer patients, the drug achieves an average mucosal concentration of 0.41 mmol/kg in the colon, with some individuals peaking at 1.87 mmol/kg [138]. As mentioned earlier, metformin treatment at 125 mg/kg (equivalent to 600 mg/average size individual of 60 kg in humans) in a pancreatic cancer mouse metastatic model effectively inhibited liver metastasis. This may be due to the TME presence in vivo, which exerts anti-tumor effects both directly killing tumor cells and indirectly through the TME, as compared to high-dose treatment in vitro.

The complex pharmacokinetic dynamics and microenvironment directly drive the notable discrepancies between preclinical models and actual human clinical outcomes. While homogeneous, well-controlled murine models demonstrate highly promising therapeutic TME reprogramming, their direct clinical translation is frequently blunted by extensive patient genomic, age-related, and immunological heterogeneity. Simplified preclinical evaluation frameworks, such as 3D microtumor co-cultures, inherently omit the dynamic immune and inflammatory components of the intact human microenvironment. When these diverse microenvironment components are absent, long-term metformin exposure can even induce paradoxical pro-tumor progression, as increasing TAMs infiltration and its S100A9 expression, and then driving EMT through the AMPK-CEBP/β-S100A9 axis alteration. Consequently, further investigations into the long-term effects and comprehensive pharmacokinetics of metformin, coupled with more sophisticated preclinical models for rigorous validation, remain imperative to successfully bridge the translational gap.

### 5.2. Combination Therapy of Metformin with TME Inhibitors

Most concerns regarding metformin involve the tumor cells themselves, ignoring the local microenvironment around them. However, it is becoming increasingly important that the most effective approach for cancer treatment involves therapies targeting both tumor cells and the TME [139]. Based on metformin’s function in immunity, tumor angiogenesis, metabolism and TME reconstruction, we focused on metformin in combination with these clinical inhibitors. For instance, Pazopanib, an inhibitor of the VEGF receptor tyrosine kinase, has demonstrated significant anti-tumor effects in lung cancer, but its drug resistance and toxicity have limited its application. However, the combination with metformin augments its anti-tumor efficacy by simultaneously targeting proliferative, angiogenic, and immunogenic signaling pathways in TME [140]. Furthermore, combined administration of the SLC25A1 citrate transporter inhibitor CTPI2, complex I inhibitor IACS-010759 and metformin has exhibited prominent in vivo inhibitory effects on tumor growth in acute myeloid leukemia [141]. In addition, immunotherapy has become a mainstay of cancer treatment in many malignancies, and combining therapies with immunotherapy has been vital for anti-tumor treatment. A non-alcoholic steatohepatitis study showed that metformin treatment rescues CD8+ T cell responses to immune checkpoint inhibitor therapy [142]. Therefore, metformin, as a critical combination therapy drug with immunotherapy, is important for cancer treatment. However, its combination with other tumor microenvironment inhibitors such as immune checkpoints, TGF-β, and CD39/CD73/A2aR inhibitors has not yet been elucidated, and we look forward to improving tumor treatment efficacy in the future.

Interestingly, metformin’s experimental anti-tumor effects contradict some clinical trials. For example, in a phase II study (ClinicalTrials.gov NCT03800602), nivolumab and metformin were well tolerated in patients with metastatic microsatellite stable colorectal cancer but had no evidence of efficacy [91]. Its combination with immune checkpoint inhibitors may potentially correlate with inferior prognoses in cancer patients in the clinical evidence [143]. However, other research also reported that metformin may improve lung cancer-specific clinical outcomes in obese and overweight lung cancer patients and enhance immunotherapy efficacy in this growing population [144]. These conflicting metformin clinical outcomes may stem from patient heterogeneity, such as metabolic profiles and specific TMEs in different cancer types. To address this discrepancy, future clinical applications should prioritize biomarker-driven patients, specifically metabolic status and immune profiles, to identify the subpopulations most likely to benefit from these combination therapies.

## 6. Conclusions and Prospects

While the traditional paradigm attributes the anti-tumor effects of metformin primarily to its direct action on tumor cells, an accumulating body of evidence supports the notion that it is the reprogramming of the TME that plays a requisite role in this process. We have elaborated on the precise regulation of various TME components using metformin. Specifically, it disrupts the metabolic support for tumor cells by regulating the metabolism of lactate, lipids, and amino acids, thereby influencing the functions of other cells within the TME. It also mediates macrophage polarization and enhances the functions of T cells, B cells, NK cells and DCs, thereby boosting the anti-tumor immune response. By degrading the ECM and inhibiting CAFs’ activity, metformin weakens the physical barriers of the tumor and improves drug penetration and immune cell infiltration. In addition, this review highlights the synergistic effects and crosstalk between different components mediated by metformin. Metformin indirectly influences tumor progression by regulating AMPK and non-AMPK mechanisms to mediate these changes via the TME, providing new insights for its anti-tumor application.

It has been shown that metformin may have anticancer and anti-diabetic activities through the classical AMPK signaling pathway and AMPK-independent pathways, such as mGPDH. This is primarily because the metabolic alterations in glycolysis and OXPHOS during its anti-diabetic action crosstalk with intracellular tumoral mechanisms, ultimately exerting its anti-tumor effects. This possibly explains why cancer patients with diabetes may experience improved prognoses following long-term metformin therapy.

The clinical application of metformin still faces several issues and challenges. Currently, the dosage required to exert anti-tumor effects in preclinical studies often reaches the mM level, which is significantly higher than that of conventional small-molecule drugs. This raises the question of whether the tumor-suppressive effect is merely a result of high dosage and whether such high-dose treatment might affect the surrounding normal cells. Additionally, metformin concentration potentially attenuates at the tumor site following systemic absorption, requiring high doses to compensate. Therefore, future studies should optimize drug delivery methods, such as direct intratumoral intervention. Modifying metformin to enhance its tumor targeting and sensitivity, such as mitochondrial-targeted analogs or TME-responsive nanocarriers, represents a promising direction for future research. Furthermore, as monotherapy, its anti-tumor activity is typically insufficient to reverse the progression of advanced tumors. Therefore, employing metformin as an adjuvant to radiotherapy, chemotherapy, and immunotherapy represents the preferred strategy for its clinical application. Last but not least, due to patient heterogeneity, identifying predictive biomarkers from individual gene expression profiles is essential to enable personalized metformin therapy. Although current Phase III trials for early breast cancer and combination trials with nivolumab for colorectal cancer have shown improved patient prognoses, more clinical trials combining metformin with TME inhibitors are needed to confirm its efficacy as an adjuvant therapy. Thus, a thorough elucidation of the molecular mechanisms by which metformin reprograms the TME will facilitate the identification of novel therapeutic targets and the development of more effective combination therapies.

## Figures and Tables

**Table 1:** Immune regulation of metformin in the tumor microenvironment: Effects on immune cell function and mechanism.

Immune Cell Types	Mechanism	Function	Tumor Types	Refs.
T Cells	JNK activation promotes infiltration; inhibiting mitochondrial complex I decreases ROS; AMPK activation induces PD-L1 degradation	Promotion: functional TILs; hypoxia tolerance; CD8+ /Treg ratio; T cell cytotoxicity; CD8+ T cell proliferation and adaptability Inhibition: exhausted T cells; apoptosis; tumor growth	Triple-negative breast cancer; melanoma; colon adenocarcinoma	[2,3,13,32,33]
B Cells	Target OXPHOS blocks metabolic reprogramming; decrease Germinal center B cells inhibit differentiation	Promotion: overcomes ibrutinib resistance; alleviates immunosuppression	Diffuse large B-cell lymphoma	[4,14,34,35]
TAMs	AMPK activation decrease NF-κB; AMPK-CEBP/β activation increase S100A9	Promotion: IL-10; TAMs polarization and infiltration Inhibition: TNF-α, IL-1β); pro-tumor phenotype	Breast cancer; lung adenocarcinoma	[15,16,36]
NK Cell	AMPK/p53 activation increase NKG2D ligands; decrease PD-L1 relieves inhibition	Promotion: tumor recognition/killing Inhibition: PD-1-mediated suppression	Diffuse large B-cell lymphoma	[19,20]
DCs	Increase eATP; induces ICD releases DAMPs	Promotion: maturation/antigen presentation; activates CD8+ T cells; tolerogenic phenotype	Ovarian cancer	[21,22,23,24,37]
MDSCs	Decrease CD39/CD73 expression; antagonizes PGE2 promote reprograms immunosuppression	Promotion: reverses immunosuppression Inhibition: Adenosine production	Ovarian cancer; non-small cell lung cancer; prostate cancer	[28,29]
TANs	Promotes N1 polarization; nanoparticle delivery boosts radiosensitivity; decreases ATP production	Promotion: recruits T/NK cells; direct tumor killing Inhibition: distant metastasis	/	[31]

**Table 2:** Molecular mechanisms of metformin within TME in a variety of tumors.

Signaling Pathway	Tumor Types	Mechanisms	Targets	Impact on the Tumor	Refs.
AMPK Pathway	Esophageal squamous cell carcinoma; colorectal cancer	Low-dose: Lysosomal AMPK activation; High-dose: Mitochondrial complex I inhibition	Direct: Mitochondrial complex I, Lysosomal pen2; Indirect: adenosine (metabolism)	Tumor cells: inhibition: proliferation, invasion, and migration TME: promotion: anti-tumor immune effects	[94,95]
STAT3 Pathway	Glioblastoma	Blocks IL-6/JAK/STAT3 axis	Indirect: IL-6 (inflammation)	Tumor cells: inhibition: proliferation and migration TME: inhibition: immunosuppressive effect	[96]
TGF-β Pathway	Pancreatic cancer	Block SMAD/non-SMAD signaling activation	Indirect: TGF-β (immune)	Tumor cells: inhibition: EMT and migration TME: inhibition: morphological changes	[97]
HIF-1α Pathway	Hepatocellular carcinoma; acute myeloid leukemia; renal carcinoma; colorectal cancer	Increase PHD-mediated degradation; inhibit PI3K/MAPK decrease HIF-1α synthesis; decrease miR-26a	Indirect: HIF-1α (vessel)	Tumor cells: promotion: autophagy and apoptosis inhibition: EMT TME: inhibition: collagen deposition; adhesion stability; angiogenesis	[5,98,99,100]
PI3K/Akt/mTOR Pathway	Lung adenocarcinoma; gastric cancer; endometrial cancer	AMPK phosphorylates p85α decrease PI3K; decrease PD-L1 expression	Indirect: PI3K (CAFs)	Tumor cells: inhibition: chemotherapy resistance, proliferation, and tumor growth TME: inhibition: immunosuppressive effect	[66,101,102]
JNK Pathway	TNBC; bladder cancer	Promote JNK phosphorylation and then increase TIL infiltration; suppress CAFs	Indirect: JNK (immune)	Tumor cells: promotion: apoptosis inhibition: proliferation and metastasis TME: promotion: anti-tumor immune effects	[3,103]
Other Pathway	Thyroid cancer; TNBC	Inhibit mGPDH activity; competitive binding to HMGB1	Direct: mGPDH; HMGB1 (inflammation)	Tumor cells: inhibition: proliferation and EMT TME: inhibition: inflammatory responses	[104,105]

## Data Availability

No new data were created or analyzed in this study.

## Abbreviations

TME: tumor microenvironment; AMPK: AMP-activated protein kinase; HIF-α: hypoxia-inducible factor 1α; ECM: extracellular matrix; JNK: c-Jun N-terminal kinase; CAFs: cancer-associated fibroblasts; IL: interleukin; ROS: reactive oxygen species; TILs: tumor-infiltrating lymphocytes; MDSCs: myeloid-derived suppressor cells; Tregs: regulatory T cells; EGR1: early growth response factor 1; OXPHOS: oxidative phosphorylation; TGF-β: transforming growth factor-β; TAMs: tumor-associated macrophages; NF-κB: nuclear factor-kappaB; TNF-α: tumor necrosis factor alpha; EMT: epithelial–mesenchymal transition; NK: Natural killer; IFN: interferon; mRNA: messenger RNA; STAT3: Signal Transducer and Activator of Transcription3; DCs: dendritic cells; eATP: extracellular ATP; TANs: tumor-associated neutrophils; VEGF: vascular endothelial growth factor; MMPs: matrix metalloproteinases; miRNA: microRNAs; LDHB: lactate dehydrogenase B; GLUT: glucose transporter; FFAs: free fatty acids; IFN: immune interferon; AP-1: activating protein-1; GPR81: G-protein-coupled receptor 81; RNS: reactive nitrogen species; mROS: mitochondrial ROS; GABA: γ-aminobutyric acid; LKB1: liver kinase B1; PHDs: prolyl hydroxylases.
